# TGF-β Mediated Immune Evasion in Cancer—Spotlight on Cancer-Associated Fibroblasts

**DOI:** 10.3390/cancers12123650

**Published:** 2020-12-05

**Authors:** Parisa Ghahremanifard, Ayan Chanda, Shirin Bonni, Pinaki Bose

**Affiliations:** 1 Department of Biochemistry and Molecular Biology, Cumming School of Medicine, University of Calgary, Calgary, AB T2N 4N1, Canada; parisa.ghahremanifar@ucalgary.ca (P.G.); achanda@ucalgary.ca (A.C.); sbonni@ucalgary.ca (S.B.); 2 Arnie Charbonneau Cancer Institute, Cumming School of Medicine, University of Calgary, Calgary, AB T2N 4N1, Canada; 3 Department of Oncology, Cumming School of Medicine, University of Calgary, Calgary, AB T2N 4N1, Canada; 4 Ohlson Research Initiative, Cumming School of Medicine, University of Calgary, Calgary, AB T2N 4Z6, Canada

**Keywords:** CAF, TGF-β, tumor immune evasion, immunotherapy

## Abstract

**Simple Summary:**

The different components surrounding a tumor are collectively known as the tumor microenvironment (TME). The transforming growth factor-beta (TGF-β) signaling pathway is activated in the TME and the tumor, leading to alteration in the composition of the TME that favors tumor growth and aggressiveness. A major component of the TME, called Cancer-Associated Fibroblasts (CAFs) help the tumor grow and escape destruction by the host immune system. TGF-β signaling and CAF-associated alterations in the TME may also predict the response to cancer immunotherapy. Whether these changes in the TME are targetable alone or in combination with TGF-β inhibition is now being tested in the clinic.

**Abstract:**

Various components of the tumor microenvironment (TME) play a critical role in promoting tumorigenesis, progression, and metastasis. One of the primary functions of the TME is to stimulate an immunosuppressive environment around the tumor through multiple mechanisms including the activation of the transforming growth factor-beta (TGF-β) signaling pathway. Cancer-associated fibroblasts (CAFs) are key cells in the TME that regulate the secretion of extracellular matrix (ECM) components under the influence of TGF-β. Recent reports from our group and others have described an ECM-related and CAF-associated novel gene signature that can predict resistance to immune checkpoint blockade (ICB). Importantly, studies have begun to test whether targeting some of these CAF-associated components can be used as a combinatorial approach with ICB. This perspective summarizes recent advances in our understanding of CAF and TGF-β-regulated immunosuppressive mechanisms and ways to target such signaling in cancer.

## 1. Introduction

Cancer cells do not exist in isolation but cohabit with non-transformed cell types and non-cellular components within a milieu generally referred to as the tumor microenvironment (TME), [1]. Specifically, the TME, in addition to cancer cells includes the extracellular matrix (ECM), secreted factors, fibroblasts, infiltrating immune cells, and tumor vasculature [2]. Recently, several reports have focused on the role of diverse TME components in tumorigenesis, progression, and metastasis. It is becoming increasingly apparent that the tumor cells co-operate with other components of the TME to form an altered ecosystem that maintains and propagates the malignant state. The establishment of an immunosuppressive niche to evade immune surveillance is a hallmark of cancer [3]. This immunosuppressive state is achieved by the intricate and continuous cross-talk between tumor cells and other stromal components of the TME, which results in the downregulation of antigen presentation, elevated expression of surface immune-inhibitory molecules, and secretion of immunosuppressive factors [4].

## 2. TGF-β and Suppression of Immune Responses in the TME

The transforming growth factor beta (TGF-β) is a pleiotropic cytokine that affects organismal development and homeostasis through its role in cell proliferation, differentiation and morphogenesis [5,6]. The TGF-β ligand family includes over 30 members in humans and their corresponding receptors are the sole Ser/Thr kinase receptors known in humans. Ligand binding leads to the phosphorylation of the type I receptor by type II, and downstream signal propagation by SMAD transcription factors [7,8,9]. Non-canonical TGF-β signaling that is independent of SMAD activation has also been reported [10]. However, TGF-β signaling involving both SMAD and non-canonical pathways can be hijacked during neoplastic transformation to promote tumor cell proliferation, epithelial-to-mesenchymal transition (EMT), and immunosuppression [9,11]. One mechanism by which TGF-β facilitates the establishment of an immunosuppressive TME is by inducing the formation of cancer-associated fibroblast (CAF) leading to increased ECM production within the TME [12]. This idea is supported by evidence of the increased TGF-β concentration in the TME, which is believed to be maintained by the tumor cells, CAFs, infiltrating immune cells and vascular cells, via autocrine or paracrine signaling [13]. Additionally, during tumorigenesis, activation of matrix metalloproteinases (MMPs) might result in the release of active TGF-β from precursor forms trapped within the ECM, leading to increased TGF-β signaling in cells of the TME [14]. Although TGF-β has multiple functions in the TME, this commentary will focus on its roles in modulating tumor immunity.

TGF-β is a well-characterized immunosuppressive cytokine that can modulate the functions of immune cell populations within the TME [15,16]. For example, TGF-β inhibits effector T cell activation by reducing Ca_2_^+^ influx, thus preventing the expression of specific transcription factors, including the nuclear factor of activated T cells (NFATc), T-bet, and GATA-3, which regulate T cell proliferation and differentiation [17]. Additionally, TGF-β collaborates with IL-2 to induce the expression of the transcription factor FOXP3 in naive CD4^+^ T cells, which in turn leads to their conversion to regulatory T (T_reg_) cells [18]. In the presence of IL-2, TGF-β induced activation of SMAD proteins leads to NFAT recruitment to the *FOXP3* gene promoter, which induces the expression of FOXP3 mRNA [14,19]. TGF-β also suppresses dendritic cells (DCs), which are the primary antigen-presenting cells (APCs) in the immune system. TGF-β inhibits antigen presentation by suppressing the expression of major histocompatibility complex class II (MHCII) [20,21,22]. Interestingly, it has been suggested that cancer cells promote the expression and activation of TGF-β in DCs, thus leading to immune evasion and tumor growth [23,24]. Another major consequence of increased TGF-β signaling is the upregulation of the transcription factor, inhibitor of differentiation 1 (Id1), which subverts the cellular differentiation program from DCs to immature myeloid-derived suppressor cells (MDSC) that are immunosuppressive in various tumor types [25,26]. Finally, TGF-β signaling has been found to inhibit the development, differentiation, and activation of cytolytic natural killer (NK) cells [14,27,28]. Several studies have elucidated the distinct mechanisms of TGF-β-mediated NK cell suppression, including alteration of the epigenetic makeup of these cells and upregulation of the mTOR signaling axis [29,30]. TGF-β also suppresses the secretion of interferon γ (IFNγ) by NK cells in a SMAD3-dependent manner, which is essential for tumor suppression by CD4^+^ TH1 cells [31].

## 3. The Origin, Function, and Heterogeneity of Cancer-Associated Fibroblasts

CAFs are myofibroblasts found within the TME that produce high levels of collagen and other ECM proteins, growth factors and cytokines (including TGF-β) that can remodel the ECM so that it favors tumorigenesis [32]. It is believed that tumor-secreted factors, including TGF-β, act on tumor-suppressive fibroblasts to induce their conversion to CAFs [33,34]. The presence of CAFs has been reported in several different solid tumors including breast, lung, pancreatic, colorectal, and gastric cancers. In contrast, brain, ovarian and renal carcinomas are characterized by very low CAF prevalence [35]. CAFs are identified by several cellular markers including α-smooth muscle actin (αSMA), S100A4/fibroblast specific protein 1 (FSP-1), fibroblast activation protein (FAP), tenascin-C, periostin, desmin, platelet-derived growth factor (PDGFR)-α, PDGFR-β, Thy-1, podoplanin, integrin β1, caveolin-1, collagen 11-α1, microfibrillar-associated protein 5, and asporin, which are present in different combinations across tumor types [35,36,37,38]. CAFs perform diverse functions in the TME that have both pro- and anti-tumorigenic effects. CAFs are able to modulate the metastatic potential of cancer cells by laying and remodeling the ECM, expressing cytokines and growth factor, regulating angiogenesis, and modulating the treatment response by impeding drug delivery to the tumor. 

Although a majority of CAFs are thought to originate from resident fibroblasts, it has been suggested that bone marrow-derived mesenchymal stem cells (MSCs), adipocytes, pericytes, and endothelial cells may also give rise to a significant proportion of these cell populations [39,40]. More recent work has focused on the possibility that distinct CAF subtypes may reside within the TME and their underlying heterogeneity may reflect the complex and specific roles that CAFs play in cancer progression and immune evasion. Using single-cell RNA sequencing (scRNA-seq), Puram et al. profiled a large number of CAFs from head and neck tumors and found that they could be partitioned into two main subsets based on the expression of immediate early response genes, mesenchymal markers, ligands and receptors, and ECM genes [41]. Intriguingly, Bartoschek et al. correlated the origins of breast cancer CAFs to their function. In particular, breast cancer CAFs could be segregated into four subpopulations, vascular (vCAFs), cycling (cCAFs), matrix (mCAFs), and developmental (dCAFs) based on their distinct gene expression profiles as determined by scRNA-seq analyses. Based on histological localization, it was inferred that mCAFs are derived from resident fibroblasts, while vCAFs and dCAFs originate from vascular sites and malignant cells, respectively [40]. Similarly, Sebastien et al. identified six different CAF populations in triple negative breast cancer using scRNA sequencing [42]. Two additional studies that focused on pancreatic ductal adenocarcinoma (PDAC), classified CAFs into myofibroblastic (myCAFs), inflammatory (iCAFs) and antigen-presenting (apCAFs) subtypes [42,43,44]. Comparing the studies on breast cancer and PDAC showed that vCAFs and myCAFs share common characteristics such as they reside close to cancer cells and express PDGFR-β, whereas iCAFs and mCAFs express PDGFR-α. MyCAFs produce high levels of α-SMA, are associated with a high contractile phenotype, and actively promote ECM remodeling. On the other hand, iCAFs and apCAFs are associated with immune suppression through the expression of interleukin 6 (IL-6) and major histocompatibility complex class II (MHCII), respectively [40,42,43,44]. Interestingly, apCAFs do not express co-stimulatory molecules like CD80, CD86, and CD40; so, although they express MHCII, they cannot activate CD4^+^ T cells [42,44]. These results suggest that CAF heterogeneity might represent distinct lineages and diverse immune-associated functions in the TME that warrant further investigation [44]. The salient features of the different CAF subtypes discussed in this section are summarized in Table 1. 

One of the major effects of TGF-β signaling is the promotion of the cellular transdifferentiation processes, the epithelial–mesenchymal transition (EMT) and endothelial–mesenchymal transition (EndMT) through which fibroblastic cells are formed from epithelial and endothelial cells, respectively [45,46]. Recently, Calon et al. and Caja et al. have reviewed the literature that highlights the upregulation of TGF-β signaling in the TME leading to transdifferentiation of different cell types into CAFs, which confirms the idea that CAFs originate from multiple sources and TGF-β plays a major role in this process [47,48]. The TGF-β signaling axis is also an important mediator of crosstalk between cancer cells and CAFs, and concurrently reprograms CAF metabolic states that augment their ability to thrive in a TME with pronounced oxidative stress [47,48]. However, the control of the fate of CAF by TGF-β is extremely complex with the activation of different Smad effectors or non-Smad pathways dictating the cellular effects and final fibroblast phenotype seen in cancer and fibrotic diseases [49,50]. In a study using skin fibroblasts and melanoma cells, it was observed that paracrine TGF-β secreted by the tumor cells promotes the expression of miR21 in the fibroblasts that suppress Smad7 translation, and finally, CAF formation [51]. Interestingly, in another study using a bladder cancer model, the paracrine TGF-β signaling from tumor cells to fibroblasts was found to be mediated by exosomes [52]. Finally, different long non-coding RNAs (lnc-RNAs) have been shown to be downstream effectors of canonical TGF-β/Smad2-3-4 signaling mediated tumor cell-CAF crosstalk with implications in the growth and metastasis of breast [33,34,53], bladder [54] and oral carcinomas [55].

## 4. The Role of CAF-Associated ECM in Immune Evasion and ICB Resistance

Based on the extent of immune cell infiltration into the tumor, the TME may be characterized as “immune deserts” or “immune inflamed”, and the tumor itself is designated as “cold” or “hot”, respectively. Immunologically hot tumors are distinguished by proinflammatory cytokine production and T cell infiltration, making them a prime target of immunotherapy [56]. Recent studies have focused on turning cold tumors into hot ones by altering the TME in order to unleash the power of ICB against these tumors as well [57]. Promotion or suppression of T-cell activation is facilitated by a class of biomolecules called immune checkpoint proteins [58]. Two key immune inhibitory checkpoints known as cytotoxic T-lymphocyte antigen 4 (CTLA-4) and programmed cell death protein 1 (PD-1) are receptors on the surface of T-cells that suppress immune responses and are specifically activated in cancers. Treatment with therapeutic antibodies designed against CTLA-4, PD-1, and its ligand PD-L1 is referred to as immune checkpoint blockade (ICB). ICB has revolutionized cancer treatment by harnessing the inherent capacity of the immune system to eliminate tumor cells locally as well as those that have metastasized, thus leading to durable responses and cures in some patients [59]. In immune hot tumors such as melanomas, ICB has led to significant improvements in mortality rates with a majority of patients responding to this treatment [58,59]. In contrast, ICB has been less effective in other solid tumors including sarcomas, brain and pancreatic cancers. This raises key questions regarding the mechanisms underlying such differences in response. It is becoming clear that responsiveness to ICB could be dependent on tumor cell-intrinsic and -extrinsic factors. These factors are thought to ultimately result in the decreased production of tumor-antigen specific CD8^+^ T-cells and decreased clonal expansion to effector T cells and memory T-cells, that together can result in the immune-destruction of tumor cells. Recently, the various mechanisms and mediators of ICB failure have been extensively discussed by Jenkins et al. and Kalbasi et al. [60,61]. Interestingly, the ability of TGF-β to promote immune exclusion, as discussed in the next section, suppresses the effect of ICB therapy for otherwise responsive hot tumors [62]. Here we will focus on a key regulator of ICB resistance and immune evasion in tumors mediated by TGF-β and CAFs.

**Figure 1:**
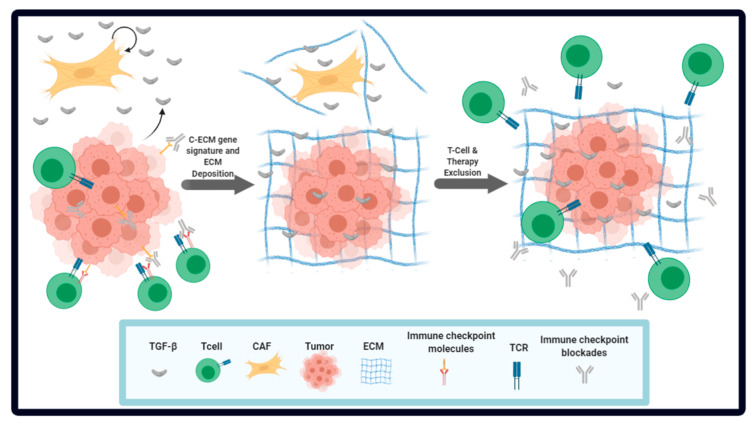
Tumor microenvironment-associated cells and extracellular components as regulators of tumor formation, survival and progression: The schematic depicts how the interplay between the tumor cells and the surrounding stroma cells including cytotoxic T-cells and cancer-associated fibroblasts (CAFs) helps in promoting tumor cell proliferation and survival mainly through evasion of attack by the immune system. Recent data support the idea that CAFs play a key role in immune evasion. In particular, tumor and mainly CAFs promote the synthesis, secretion, and activation of TGF-β in the tumor microenvironment. TGF-β, in turn, acts on CAFs, leading to extracellular matrix (ECM) remodeling to limit access of cytotoxic T-cells and immune checkpoint blockade to the tumor.

We and others have interrogated the role of TME components, and specifically, the role of CAFs in immune evasion [63,64,65,66]. Mariathasan et al. used samples from 298 patients with metastatic urothelial cancers treated with an anti-PD-L1 antibody. The lack of response in the non-responders was attributed to active TGF-β signaling in CAFs, which lead to increased ECM deposition in the TME, and thus to the exclusion of CD8^+^ T-cells. In a mouse model for urothelial carcinoma, combinatorial therapy with a TGF-β antagonist and anti-PD-L1 antibody led to potent tumor regression and suppression of metastatic spread [63]. Similarly, Tauriello et al. showed that colorectal cancer metastasis could not be suppressed by ICB due to high expression of TGF-β in the cancer stroma. However, co-treatment with a TGF-β inhibitor and ICB was highly efficacious in reducing metastatic spread and the number of metastases [64]. We used publicly available TCGA RNA-seq data from 15 different tumor types and identified an adaptive mechanism in tumors that utilizes ECM-related genes to promote immune evasion and immunotherapy resistance. We found that deregulation of ECM genes was a hallmark of cancer. A class of ECM molecules were differentially expressed across cancer types, suggesting a pan-cancer signature; we called these genes cancer-associated ECM (C-ECM) genes. Interestingly, 48 out of 58 C-ECM genes that we identified were also part of the cancer matrisome (total components of the ECM) in another proteomics-based study [67]. We identified CAFs as the main type of cells responsible for C-ECM related changes in the TME and did not find other ECM-infiltrating cells such as leukocytes [65], which corroborates their role in the production of ECM-associated molecules [32]. Interestingly, only the expression of upregulated C-ECM genes was correlated with significantly worse prognosis [65], but the expression of these genes was found to correlate with markers for immunologically hot tumors such as high mutational burden, Class I neoantigen abundance and microsatellite instability. This suggested the presence of an adaptive response in these hot tumors that helps them evade immune detection. Consistent with this hypothesis, the top quartile of C-ECM up genes was enriched for gene ontology terms associated with inflammatory processes and adaptive immune responses [65]. On further investigation, we observed a significant increase in the concentration of TGF-β in tumors expressing high amounts of C-ECM-up genes. Moreover, we identified TGF-β signaling as a driver of the C-ECM-up signature in CAFs. We also identified non-silent mutations in several genes associated with cancer progression and TGF-β signaling including *TP53*, *SMAD4*, *BRAF* and *c*-*MYC* in C-ECM-up high tumors [65]. Our data also indicate that in ICB-treated patient cohorts, the C-ECM-up signature was significantly upregulated in non-responders compared to responders. We further developed a 19-genes predictive signature of ICB therapy response comprising C-ECM-up genes that outperformed conventionally used predictors such as cytolytic activity, a T-cell inflamed signature and mutational load. The 19-gene C-ECM-up signature also outperformed a TGF-β signature and CAF abundance for predicting ICB response. The key points from the above studies are illustrated in Figure 1. Overall, we and others have uncovered CAF-associated immune evasion mechanisms that might be helpful in predicting responses to ICB. The ECM-derived ICB response predictive signature requires further prospective validation.

CAFs can suppress immune cell activity in the TME in multiple ways including the production of immunosuppressive cytokines and immune checkpoint ligands, anti-tumor CD8^+^ T-cells exclusion, and by modulating the functional differentiation of tumor-infiltrating inflammatory cells [68,69]. The immunosuppressive milieu of the TME is partially promoted by the increased concentration of different ligands that recruit myeloid cells, which can be altered by CAFs to become tumor-promoting counterparts [70,71,72,73,74]. αSMA-positive CAFs are a significant source of TGF-β in the tumor microenvironment that regulate the immunosuppressive nature of the TME, especially by regulating NK cells [75]. CAFs may also recruit T_reg_ cells in a TGF-β-dependent manner [69]. Finally, CAFs alter the composition of the ECM by secreting proteins and other ECM-related molecules, like collagen and hyaluronan, that may act collectively as physical barriers to the infiltrating cytotoxic lymphocytes and therapeutic interventions [2,76].

## 5. Novel Therapeutic Insights for Attaining an Immune-Favorable TME

Following the reports described above, several studies have confirmed the correlation between a CAF-mediated and TGF-β-regulated ECM-based signature and ICB response/patient outcome in diverse tumor types [77,78,79,80]. In such a scenario, tumors with an elevated C-ECM signature will benefit from a combinatorial therapy regimen consisting of TGF-β signaling inhibition and ICB [81]. A clinical trial reported by Feun et al. (NCT02658019) found that TGF-β levels in baseline plasma positively correlated with worse outcomes in pembrolizumab (a PD-1 blocking antibody)-treated advanced hepatocellular carcinoma patients [82]. Several trials are evaluating the efficacy of dual TGF-β inhibition and ICB in different tumors [83] and show great promise for the treatment of advanced malignancies.

However, recent reports from our lab and others suggest that a strategy of pushing the tumor microenvironment towards a more “normal-like” ECM contexture might be more efficacious than targeting TGF-β itself. Inhibiting the CAF-ECM response should improve the efficacy of ICB in tumors where immune cells are excluded due to the physical barrier formed by the ECM around the tumor mass, irrespective of the tumor type. Indeed, several clinical trials are underway that are investigating the effects of different drugs that disrupt the activation of CAFs or deregulate CAF-induced ECM deposition [39]. Recent studies have also suggested that targeting CAF-specific genes may sensitize tumor cells to immunotherapy-based interventions [74,79]. Overall, these studies provide exciting new avenues for the treatment of immunologically hot tumor types and promote the efficacy of ICB-based drugs. 

## 6. Summary and Conclusions

In summary, TGF-β signaling and CAFs-associated mechanisms play a significant role in tumor growth, metastasis, and therapy resistance. Based on their origin and genetic makeup, different CAF subtypes have been identified and they perform diverse functions in the context of the immunologic make-up of the TME. The TGF-β signaling pathway has emerged as a master regulator of the immune contexture in the TME that orchestrates the interplay between tumor cells and CAFs, leading to changes in the ECM that excludes immune cells and possibly affecting immunotherapy responses. Targeting TGF-β signaling and ECM changes, alone or in combination, hold great promise for improving the efficacy of cancer immunotherapy.

## Figures and Tables

**Table 1:** Summary of cancer-associated fibroblast (CAF) subtypes.

Disease Model	Subtype	Features	Reference
Breast Cancer	vCAF	Derived from cells in the perivascular location. Express PDGFR-α. Produce high levels of α-SMA.	Bartoschek et al. [40] Sebastien et al. [42]
	cCAF	Similar to vCAF, except high expression of Ki67 and cell cycle genes. Thought to be vCAFs that are proliferative.	
	mCAF	Derived from resident fibroblasts. Express PDGFR-β. Gene signatures for ECM activation and EMT observed.	
	dCAF	Derived from epithelial tumor cells. Express genes related to tumor initiating cells.	
	apCAF	Express MHCII but no other co-stimulatory molecules. Also express CD74. Can activate CD4+ T cells in an antigen-specific fashion.	Sebastien et al. [42]
Pancreatic Ductal Adenocarcinoma	myCAF	Derived from pancreatic stem cells and bone marrow-derived mesenchymal stem cells. Reside close to bulk of the primary tumor. Express PDGFR-α and α-SMA similar to vCAFs identified in breast cancer.	Ohlund et al. [43]
	iCAF	Secrete inflammatory cytokines. Reside far from the tumor, possibly originating from resident fibroblasts. Express PDGFR-β similar to mCAFs identified in breast cancer.	
	apCAF	Similar to breast cancer apCAFs described above.	Elyada et al. [44]
